# Anti-Atherogenic Effects of Vaspin on Human Aortic Smooth Muscle Cell/Macrophage Responses and Hyperlipidemic Mouse Plaque Phenotype

**DOI:** 10.3390/ijms19061732

**Published:** 2018-06-11

**Authors:** Kengo Sato, Remina Shirai, Maho Yamaguchi, Tomoyuki Yamashita, Koichiro Shibata, Taisuke Okano, Yusaku Mori, Taka-aki Matsuyama, Hatsue Ishibashi-Ueda, Tsutomu Hirano, Takuya Watanabe

**Affiliations:** 1 Laboratory of Cardiovascular Medicine, Tokyo University of Pharmacy and Life Sciences, Tokyo 192-0392, Japan; ksato@toyaku.ac.jp (K.S.); s106101@toyaku.ac.jp (R.S.); s139110@toyaku.ac.jp (M.Y.); s126208@toyaku.ac.jp (T.Y.); s149064@toyaku.ac.jp (K.S.); s149021@toyaku.ac.jp (T.O.); 2 Department of Medicine, Division of Diabetes, Metabolism, and Endocrinology, Showa University School of Medicine, Tokyo 142-8666, Japan; torigoe1234@yahoo.co.jp (Y.M.); hirano@med.showa-u.ac.jp (T.H.); 3 Department of Legal Medicine, Showa University School of Medicine, Tokyo 142-8555, Japan; taka94242879@gmail.com; 4 Department of Pathology, National Cerebral and Cardiovascular Center, Osaka 565-8565, Japan; hueda@ncvc.go.jp

**Keywords:** vaspin, atherosclerosis, macrophage, vascular smooth muscle cell, *Apoe*^−/−^ mice

## Abstract

Vaspin (visceral adipose tissue-derived serine protease inhibitor) was recently identified as a novel adipocytokine with insulin-sensitizing effects. Serum vaspin levels are reported either increased or decreased in patients with coronary artery disease. Our translational research was performed to evaluate the expression of vaspin in human coronary atherosclerotic lesions, and its effects on atherogenic responses in human macrophages and human aortic smooth muscle cells (HASMC), as well as aortic atherosclerotic lesion development in spontaneously hyperlipidemic *Apoe*^−/−^ mice, an animal model of atherosclerosis. Vaspin was expressed at high levels in macrophages/vascular smooth muscle cells (VSMCs) within human coronary atheromatous plaques. Vaspin significantly suppressed inflammatory phenotypes with nuclear factor κB down-regulation in human macrophages. Vaspin significantly suppressed oxidized low-density lipoprotein-induced foam cell formation with CD36 and acyl-coenzyme A: cholesterol acyltransferase-1 down-regulation and ATP-binding cassette transporters A1 and G1, and scavenger receptor class B type 1 up-regulation in human macrophages. Vaspin significantly suppressed angiotensin II-induced migration and proliferation with ERK1/2 and JNK down-regulation, and increased collagen production with phosphoinositide 3-kinase and Akt up-regulation in HASMCs. Chronic infusion of vaspin into *Apoe*^−/−^ mice significantly suppressed the development of aortic atherosclerotic lesions, with significant reductions of intraplaque inflammation and the macrophage/VSMC ratio, a marker of plaque instability. Our study indicates that vaspin prevents atherosclerotic plaque formation and instability, and may serve as a novel therapeutic target in atherosclerotic cardiovascular diseases.

## 1. Introduction

Atherosclerosis is a chronic inflammatory disease arising from endothelial injury and accumulation of cholesterol-laden macrophage foam cells in the artery wall [1]. Macrophages play a key role in vascular inflammation by changing phenotype of pro-inflammatory (M1) or anti-inflammatory (M2) [2]. Macrophage foam cell formation is characterized by cholesterol ester accumulation that depends on the homeostatic balance among the uptake of oxidized low-density lipoprotein (LDL) via a scavenger receptor CD36, the efflux of free cholesterol controlled by ATP-binding cassette transporters A1 and G1 (ABCA1, ABCG1) and scavenger receptor class B type 1 (SRB1), and cholesterol esterification from excess free cholesterol by acyl-coenzyme A: cholesterol acyltransferase 1 (ACAT1) [3]. Cholesterol ester stored in lipid droplets can be removed from cells only after hydrolysis, to free cholesterol by neutral cholesterol ester hydrolase (NCEH) [4]. Vascular smooth muscle cells (VSMCs) contribute to the formation of atheromatous plaque by their migration, proliferation, and production of extracellular matrix (ECM) components, such as collagen, matrix metalloproteinase (MMP), fibronectin, and elastin [5]. In addition to plaque size, plaque phenotype is also important in evaluating plaque rupture [6].

Vaspin (visceral adipose tissue-derived serine protease inhibitor) was identified as a 45.2-kDa adipocytokine secreted from visceral adipose tissues of Otsuka Long-Evans Tokushima fatty rats, an animal model of type 2 diabetes with obesity [7]. Human vaspin belongs to serpin family A member 12 (serpin A12), which is coded by *SERPINA12*. Human vaspin precursor protein contains 414 amino acids that include the 395 amino acid peptide hormone and a 19 amino acid signal peptide. Human and mouse vaspin share about 81% amino acid sequence of reactive site loop identity (~62% identity in full-length), and exhibit cross-species activity [7]. Vaspin is expressed in visceral and subcutaneous adipose tissues, peripheral blood mononuclear cells, macrophage foam cells, and VSMCs [8,9,10,11]. The receptors for vaspin are still not elucidated. Vaspin is known to effect glucose-insulin homeostasis, appetite regulation, bone metabolism, and vascular health [7,12,13,14].

Vaspin inhibits the generation of reactive oxygen species (ROS) and the expression of pro-inflammatory molecules, such as interleukin 1, monocyte chemotactic protein 1, vascular cell adhesion molecule 1, intercellular adhesion molecule 1, and selectin E in human vascular endothelial cells (ECs), and monocyte-EC adhesion [15,16,17]. Vaspin inhibits platelet-derived growth factor BB-induced migration and high glucose-induced proliferation in rat VSMCs [18,19]. Vaspin enhances cholesterol efflux by ABCA1 up-regulation in THP1 monocyte-derived macrophages [20]. Administration and overexpression of vaspin ameliorates glucose tolerance, insulin sensitivity, atherosclerosis, and neointimal hyperplasia of balloon-injured carotid arteries in murine [7,21,22,23]. Several lines of clinical evidence have shown that blood vaspin levels are elevated in patients with obesity, diabetes, metabolic syndrome, and coronary artery disease (CAD) [24,25,26,27]. In contrast, other studies have shown that blood vaspin levels are decreased in the presence and severity of CAD [11,28,29,30,31].

In the present study, we assessed the expression levels of vaspin in human coronary artery lesions in CAD patients, and the anti-atherogenic effects of vaspin on the inflammatory phenotype and foam cell formation in human macrophages, as well as the migration, proliferation, and ECM production in human aortic smooth muscle cells (HASMCs) in vitro. In addition, in vivo experiments focused on the inhibitory effects of vaspin against the development of aortic atherosclerotic lesions and plaque instability in spontaneously hyperlipidemic *Apoe*^−/−^ mice, an animal model of atherosclerosis. This study was performed as translational research, i.e., it is an attempt to study a candidate therapeutic target for atherosclerosis.

## 2. Results

### 2.1. Expression of Vaspin in Human Coronary Atherosclerosis

**Figure 1:**
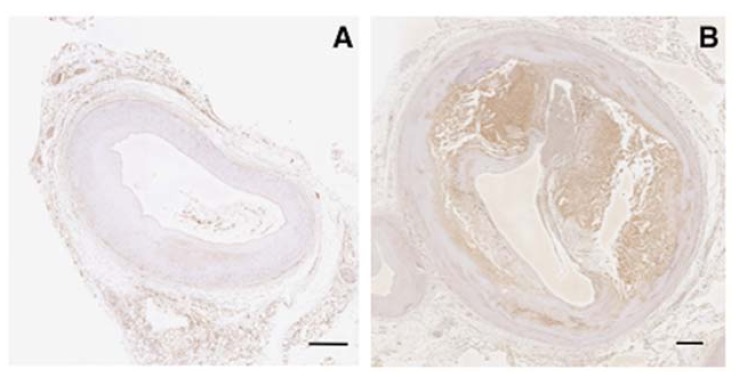
Immunohistological expression of vaspin in human coronary arteries from a non-CAD patient (male, 39 years old, dilated cardiomyopathy; (**A**)) and a CAD patient (male, 80 years old, acute myocardial infarction; (**B**)). Bar = 500 μm.

The expression of vaspin was not observed in human normal coronary arteries (Figure 1A). However, vaspin was expressed at high levels in atheromatous plaques (macrophage foam cells) and thick media (VSMCs) in human coronary arteries (Figure 1B).

### 2.2. Expression of Vaspin in Human Vascular Cells

**Figure 2:**
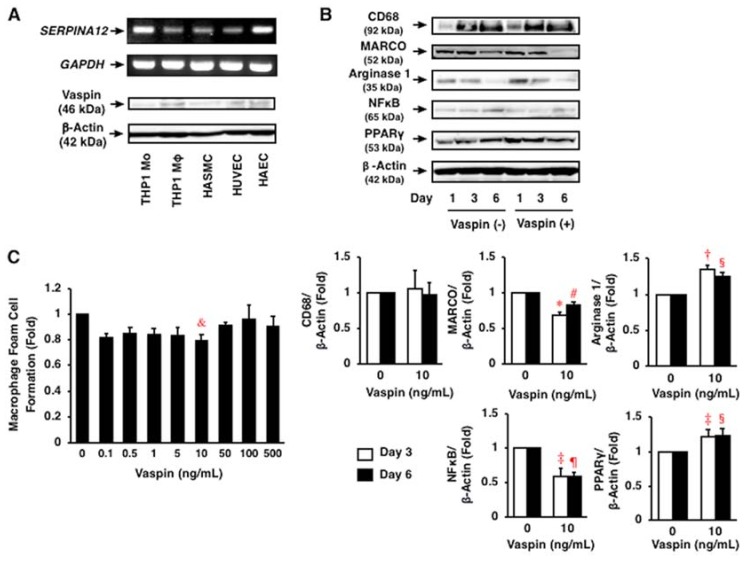
Expression of vaspin and its effects on inflammatory phenotype and foam cell formation in human macrophages. (**A**) Expression of *SERPINA12* and vaspin in human vascular cells, such as THP1 monocytes, their derived macrophages, HASMCs, HUVECs, and HAECs was assessed by RT-PCR and immunoblotting. *GAPDH* and β-actin served as loading controls. (**B**) Effects of vaspin on inflammatory phenotypes in THP1 monocyte-derived macrophages were assessed by immunoblotting (*n* = 3–4). * *p* < 0.0005, ^†^ *p* < 0.005, ^‡^ *p* < 0.05 vs. control on day 3; **^#^** *p* < 0.005, ^§^ *p* < 0.05, ^¶^ *p* < 0.0005 vs. control on day 6. (**C**) Effects of vaspin on oxidized LDL-induced foam cell formation in THP1 monocyte-derived macrophages were assessed by cholesterol esterification assay (*n* = 5). 1 fold = 5.72 ± 0.65 nmol/mg cell protein. ^&^ *p* < 0.05 vs. 0 ng/mL of vaspin.

*SERPINA12* and vaspin was abundantly expressed in THP1 monocytes, their derived macrophages, HASMCs, human umbilical vein endothelial cells (HUVECs), and human aortic endothelial cells (HAECs) at mRNA and protein expression levels (Figure 2A).

### 2.3. Effects of Vaspin on Inflammatory Phenotype in Human Monocytes/Macrophages

After 1–6 days of culture, the differentiation of THP1 monocytes into macrophages was confirmed by increased expression of CD68, a macrophage differentiation marker (Figure 2B). Vaspin (10 ng/mL) did not affect monocyte differentiation into macrophages. However, vaspin (10 ng/mL) significantly decreased the expression of MARCO, an M1 marker, and increased arginase 1, an M2 marker, through differentiation (*p* < 0.05 to *p* < 0.0005; Figure 2B). These observations indicated that vaspin shifted the macrophage phenotype overwhelmingly to M2 rather than M1, which was associated with significant changes of nuclear factor κB (NFκB) down-regulation and peroxisome proliferator-activated receptor γ (PPARγ) up-regulation (*p* < 0.05 to *p* < 0.0005; Figure 2B).

### 2.4. Effects of Vaspin on Human Macrophage Foam Cell Formation

**Figure 3:**
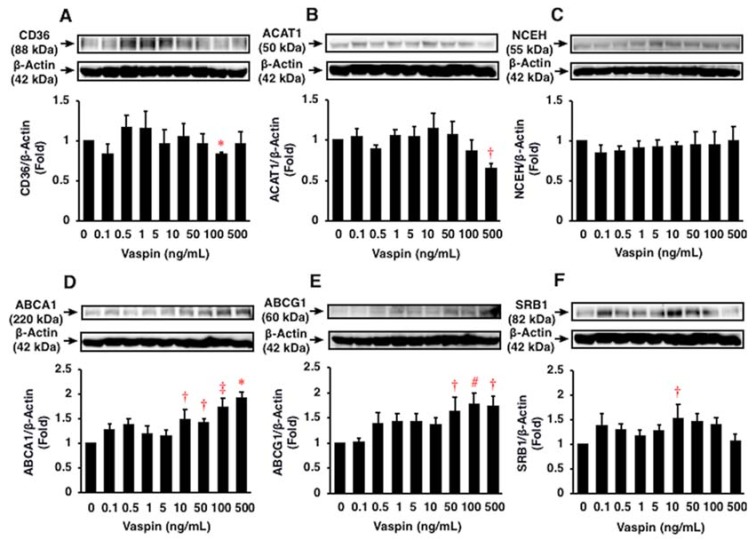
Effects of vaspin on foam cell formation related protein expression in THP1 monocyte-derived macrophages were assessed by immunoblotting. The blotted membranes were cut for reacting with different antibodies, and the blots were stripped and reprobed in the same membrane. Top: Representative results of protein expression of each CD36 (**A**), ACAT1 (**B**), NCEH (**C**), ABCA1 (**D**), ABCG1 (**E**), and SRB1 (**F**). Bottom: Densitometric data of each molecule after normalization relative to β-actin (*n* = 5–6). * *p* < 0.0001, ^†^ *p* < 0.05, ^‡^ *p* < 0.001, ^#^ *p* < 0.01 vs. 0 ng/mL of vaspin.

Vaspin suppressed oxidized LDL-induced foam cell formation by 20% at 10 ng/mL in THP1 monocyte-derived macrophages (*p* < 0.05; Figure 2C). Vaspin at 100 and 500 ng/mL suppressed the protein expression of CD36 and ACAT1 by 20% and 30%, respectively (*p* < 0.0001, *p* < 0.05; Figure 3A,B), but not NCEH (*p* > 0.05; Figure 3C). Vaspin enhanced the protein expression of ABCA1, ABCG1, and SRB1 by 1.5–2.0 fold at 10–500 ng/mL (*p* < 0.05 to *p* < 0.0001; Figure 3D–F).

### 2.5. Effects of Vaspin on Migration and Proliferation of HASMCs

**Figure 4:**
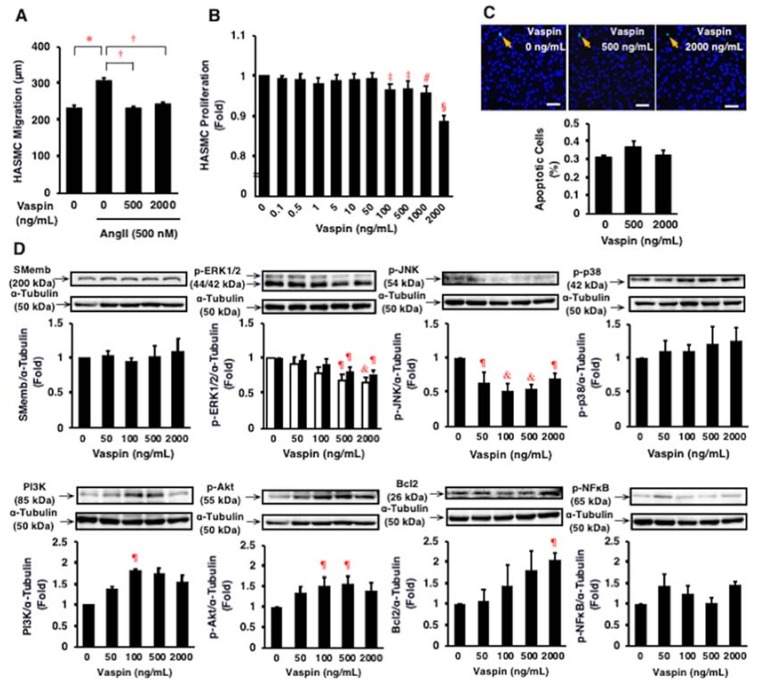
Effects of vaspin on migration, proliferation, apoptosis, and intracellular signal transductions in HASMCs. (**A**) Effect of vaspin on angiotensin II (Ang II)-induced migration was determined in 10 cells per well using a BIOREVO BZ-9000 microscope. The experiments were repeated independently 4 times. * *p* < 0.005, ^†^ *p* < 0.0001. (**B**) Effect of vaspin on proliferation was determined by WST-8 assay (*n* = 4). ^‡^ *p* < 0.05, **^#^** *p* < 0.001, ^§^ *p* < 0.0001 vs. 0 ng/mL of vapin. (**C**) Effect of vaspin on apoptosis was evaluated by detecting apoptotic cells (green) using the TUNEL method. Nuclei were co-stained with 6-diamidino-2-phenylindole (blue). The graph indicates the percentage of apoptotic cells in 3 independent experiments. Bar = 100 μm. (**D**) Relevant intracellular signals in response to vaspin were assessed by immunoblotting. The blotted membranes were cut for reacting with different antibodies, and the blots were stripped and reprobed in the same membrane. Top: Representative results of protein expression/phosphorylation of SMemb, ERK1/2, JNK, p38, PI3K, Akt, Bcl2, and NFκB. Bottom: Densitometric data of each molecule after normalization relative to α-tubulin (*n* = 4–5). ^¶^ *p* < 0.05, ^&^ *p* < 0.01 vs. 0 ng/mL of vapin (corresponding control).

Angiotensin II (500 nmol/L) significantly increased the migration of HASMCs (*p* < 0.005; Figure 4A). Vaspin (500, 2000 ng/mL) significantly suppressed the angiotensin II-induced migration of HASMCs (both *p* < 0.0001; Figure 4A). Vaspin (100–2000 ng/mL) significantly suppressed the proliferation of HASMCs (*p* < 0.05 to *p* < 0.0001; Figure 4B). However, vaspin (500, 2000 ng/mL) did not affect significantly apoptosis in HASMCs (Figure 4C).

### 2.6. Effects of Vaspin on Intracellular Signaling in HASMCs

Vaspin significantly suppressed ERK1/2 and JNK phosphorylation, but increased phosphoinositide 3-kinase (PI3K) and Bcl2 expression and Akt phosphorylation in HASMCs (*p* < 0.05 to *p* < 0.01; Figure 4D). Vaspin did not significantly alter the expression of SMemb, a proliferative marker, and p38 and NFκB phosphorylation in HASMCs (*p* > 0.05; Figure 4D).

### 2.7. Effects of Vaspin on ECM Expression in HASMCs

**Figure 5:**
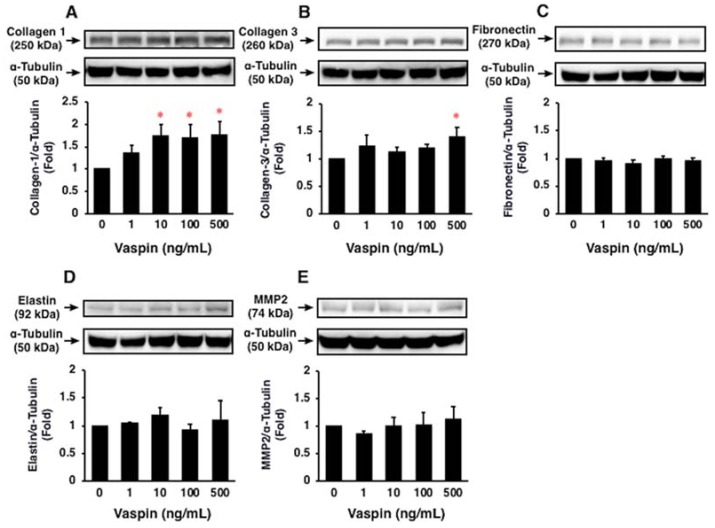
Effects of vaspin on ECM expression in HASMCs were assessed by immunoblotting. The blottted membranes were cut for reacting with different antibodies, and the blots were stripped and reprobed in the same membrane. Top: Representative results of protein expression of collagen 1 (**A**), collagen 3 (**B**), fibronectin (**C**), elastin (**D**), and MMP2 (**E**). Bottom: Densitometric data of each molecule after normalization relative to α-tubulin (*n* = 3–4). * *p* < 0.05 vs. 0 ng/mL of vaspin.

Vaspin significantly increased protein expression of collagen 1 and collagen 3 in HASMCs (both *p* < 0.05; Figure 5A,B). However, vaspin had no significant effects on protein expression of fibronectin, elastin, and MMP2 in HASMCs (*p* > 0.05; Figure 5C–E).

### 2.8. Effects of Vaspin on Atherosclerotic Lesion Development in Apoe^−/−^ Mice

Body weight significantly increased with age (17 to 21 weeks old), but did not differ significantly among 21-week-old *Apoe*^−/−^ mice infused with 3 doses of vaspin (Table 1). There were no significant differences in food intake, systolic and diastolic blood pressures, or plasma levels of total cholesterol, high-density lipoprotein (HDL) cholesterol, and non-HDL cholesterol among four groups of *Apoe*^−/−^ mice (Table 1). High-dose of vaspin significantly decreased free fatty acid level and tended to decrease fasting glucose and triglyceride levels and homeostasis model assessment of insulin resistance (HOMA-IR) in *Apoe*^−/−^ mice (Table 1). Low-dose of vaspin significantly increased both plasma insulin level and HOMA-IR in *Apoe*^−/−^ mice (Table 1).

**Figure 6:**
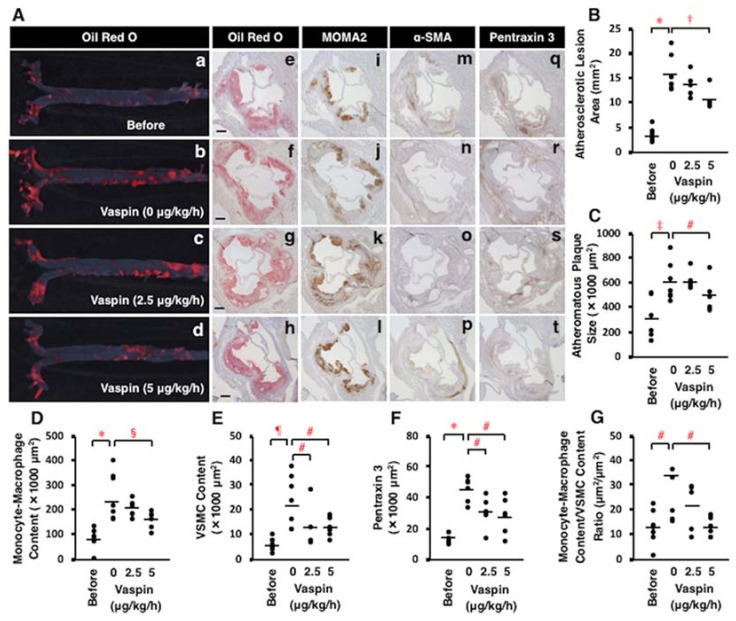
Effects of vaspin on atherosclerotic lesion development in *Apoe*^−/−^ mice. Seven mice were sacrificed before infusion (17 weeks old), and 7, 5, and 6 mice were sacrificed after a 4-week continuous subcutaneous infusion of 3 dosing rates of vaspin (0, 2.5, 5 μg/kg/h), respectively. (**A**) Atherosclerotic lesions were stained with Oil Red O on the aortic surface (**a**–**d**). Cross-sections of the aortic sinus were stained with Oil Red O (**e**–**h**), MOMA2 for monocytes/macrophages (**i**–**l**), α-SMA for VSMCs (**m**–**p**), or pentraxin 3 for vascular inflammation (**q**–**t**). Hematoxylin was used for nuclear staining. Bar = 200 μm. (**B**–**G**) Comparisons of these positive area and the ratio of monocyte-macrophage content/VSMC content within atheromatous plaques were performed among 4 groups. Bars indicate the mean values in the graphs. * *p* < 0.0001, ^†^ *p* < 0.005, ^‡^ *p* < 0.001, ^#^ *p* < 0.05, ^§^ *p* < 0.01, ^¶^ *p* < 0.0005.

Both the entire atherosclerotic lesions in aortic lumen surface and plaque size of aortic sinus wall significantly increased with age in *Apoe*^−/−^ mice (*p* < 0.0001, *p* < 0.001; Figure 6A(a,b,e,f),B,C). These were accompanied with significant increases in monocyte-macrophage infiltration, VSMC content, vascular inflammation (pentraxin 3), and the ratio of monocyte-macrophage content/VSMC content in atheromatous plaques (*p* < 0.05 to *p* < 0.0001; Figure 6A(i,j,m,n,q,r),D–G). However, 4-week infusion of high-dose vaspin significantly decreased the entire atherosclerotic lesions in aortic lumen surface and plaque size of aortic sinus wall (*p* < 0.005, *p* < 0.05; Figure 6A(b,d,f,h),B,C). Vaspin infusion at high dose or both doses significantly decreased monocyte-macrophage infiltration, VSMC content, pentraxin 3 expression, and the ratio of monocyte-macrophage content/VSMC content, a biomarker of plaque instability, in atheromatous plaques (*p* < 0.05 to *p* < 0.01; Figure 6A(j–l,n–p,r–t),D–G).

## 3. Discussion

**Figure 7:**
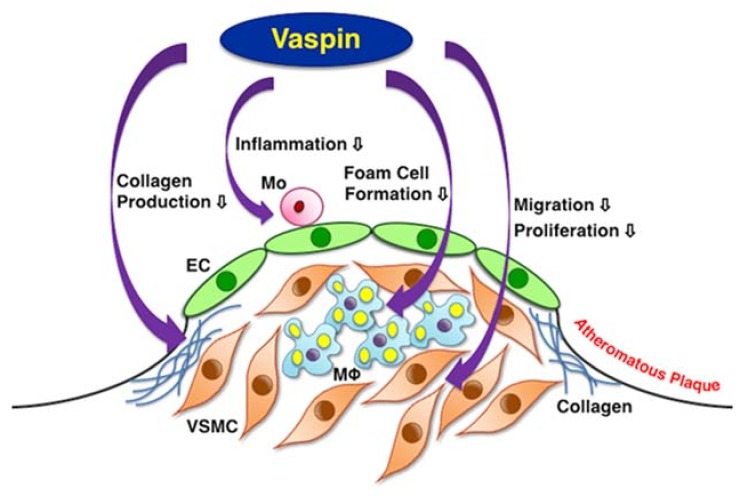
Schema of multiple anti-atherogenic effects of vaspin. EC = endothelial cell, Mo = monocyte, Mφ = macrophage, VSMC = vascular smooth muscle cell.

The present study provides comprehensive evidence that vaspin inhibits atherogenesis by suppressing the inflammatory phenotype and foam cell formation in macrophages, as well as the migration and proliferation of VSMCs (Figure 7). Vaspin also contributes to stabilizing plaque by increasing collagens and reducing the macrophage/VSMC ratio in atheromatous plaques (Figure 7). Other studies have shown that vaspin inhibits the expression of pro-inflammatory molecules in ECs and monocyte-EC adhesion [16,17]. Lin et al. have shown that vaspin attenuates atherogenesis by inhibiting endoplasmic reticulum (ER) stress-induced macrophage apoptosis in *Apoe*^−/−^ mice [22].

Vaspin transgenic mice prevented diet-induced obesity, glucose intolerance, and hepatic steatosis, while vaspin-deficient mice developed glucose intolerance associated with up-regulation of ER stress markers [21]. Vaspin improved ER stress and insulin resistance in obese mice by acting as a ligand for cell-surface glucose regulated protein 78/murine tumor cell DnaJ-like protein 1 complex via p-Akt in the liver [21]. Adenovirus vector carrying the full length of the vaspin suppressed neointimal hyperplasia of balloon-injured carotid arteries in streptozotocin-induced diabetic Wistar rats [23]. The intimal proliferation was also suppressed in cuff-injured femoral arteries in vaspin transgenic mice [23].

Several studies have shown that vaspin increases insulin secretion and ameliorates insulin resistance [7,32]. Vaspin also inhibits kallikrein 7-mediated insulin degradation by a classical serpin mechanism [33]. The present study showed that chronic infusion of high-dosing rate of vaspin (5 μg/kg/h) improved insulin resistance and decreased fasting plasma glucose level in *Apoe*^−/−^ mice. However, chronic infusion of low-dosing rate of vaspin (2.5 μg/kg/h) did not improve insulin resistance with increased fasting plasma glucose and insulin levels in *Apoe*^−/−^ mice. We speculate that the reason for this is as follows: vaspin infusion into *Apoe*^−/−^ mice may start to stimulate insulin secretin from pancreas at low doses and accelerate to ameliorate insulin sensitivity in skeletal muscle, fat, and liver in dose-dependent manner. Further studies, such as glucose/insulin tolerance test, are needed to clarify the precise mechanism.

Previous studies have shown that vaspin protects blood vessels by suppressing ROS generation and inflammation via down-regulating the NFκB pathway, and by inhibiting apoptosis via up-regulating the PI3K-Akt-endothelial nitric oxide synthase pathway in ECs [15,33,34,35]. In the present study, vaspin decreases M1 phenotype acquisition and increases the expression of the M2 phenotype associated with NFκB down-regulation and PPARγ up-regulation in monocyte-derived macrophages. Vaspin suppresses foam cell formation associated with CD36 and ACAT1 down-regulation, as well as ABCA1, ABCG1, and SRB1 up-regulation in macrophages. Vaspin suppresses the migration and proliferation via the down-regulation of ERK1/2 and JNK pathways, and increases collagen production via the up-regulation of the PI3K-Akt pathway in VSMCs. It is possible that vaspin may not induce apoptosis via activating the PI3K-Akt-Bcl2 pathway in VSMCs.

We discuss the integrity of vaspin levels in our experiments. Several studies have shown that plasma concentrations of vaspin are ~2.18 ng/mL in healthy subjects and ~0.47 ng/mL in CAD patients [29]. The concentrations of vaspin required for modulation of several responses of THP1 monocyte-derived macrophages and HASMCs were 10–2000 ng/mL in our study, and were 100–150 ng/mL for macrophage and EC responses in other studies [17,20,36]. According to our previous studies [37,38,39], atheroprotective agents are increased to counteract the development of atherosclerosis. The local levels of vasoactive agents could increase to a similar degree by the generation from vascular cells in an autocrine/paracrine fashion [40,41]. Our study shows that the expression of vaspin is increased in macrophages and VSMCs within coronary atherosclerotic lesions. However, decreased circulating blood levels of vaspin in CAD patients may be attributed to severe endothelial dysfunction due to CAD. In monocyte-derived macrophages, the adequate concentrations of vaspin differed in inducing foam cell formation and related protein expression (10–500 ng/mL). This is mostly dependent on the difference in the presence or absence of oxidized LDL. Vaspin at higher than adequate concentrations might lead to the down-regulation of the receptor and intracellular signals. The adequate concentrations of vaspin also differ between macrophages and VSMCs.

Several clinical studies have shown that circulating blood levels of vaspin are significantly increased to improve insulin resistance in patients with type 2 diabetes and metabolic syndrome [24,25,26], but decreased due to severe endothelial dysfunction in patients with CAD [11,29,30]. In patients with type 2 diabetes, vaspin levels are decreased by improvement in insulin resistance with exercise and metformin in men and women, respectively [42,43]. Vaspin levels are increased by rosuvastatin in obese patients with CAD [44]. This is attributed to the improvement effect of rosuvastatin on endothelial dysfunction in CAD patients [45].

In conclusion, the results from the present study indicate that vaspin inhibits atherogenesis by suppressing vascular inflammation, macrophage foam cell formation, and VSMC migration and proliferation. Vaspin also contributes to stabilizing plaque by increasing collagens and reducing the intraplaque macrophage/VSMC ratio. Targeting vaspin allows us to open a therapeutic window for combating atherosclerosis and related diseases, as well as for maintaining vascular health.

## 4. Materials and Methods

### 4.1. Materials

Recombinant human vaspin produced in *E. coli* was purchased from PeproTech (Rocky Hill, NJ, USA) for in vitro experiments and GenScript (Piscataway, NJ, USA) for in vivo experiments; the purity was ≥98% and >95%, respectively. Rabbit polyclonal anti-human vaspin antibody was purchased from GeneTex (Irvine, CA, USA). Angiotensin II was purchased from Sigma (St. Louis, MO, USA), and phorbol 12-myristate 13-acetate was from Wako (Osaka, Japan). HUVECs and HASMCs were purchased from Lonza (Walkersville, MD, USA) and THP1 monocytes were from Health Science Research Resources Bank (Osaka, Japan).

### 4.2. Human Coronary Artery Immunostaining

This study was deemed exempt as retrospective autopsied coronary artery specimens by the National Cerebral and Cardiovascular Center Review Board (M18-020, 27 July 2006). Written informed consent to autopsy was obtained from families. Buffered 10% formalin-fixed paraffin-embedded human coronary artery specimens from archive autopsy collections of the National Cerebral and Cardiovascular Center were used for immunohistochemistry. Serial cross-sections of coronary arteries from four male patients with CAD (aged 71–87) and three male patients with dilated cardiomyopathy (as non-CAD examples) (aged 19–39) were stained with rabbit polyclonal anti-human vaspin antibody. Immunodetection (as a secondary antibody) was performed with the Bond Polymer Refine Detection kit (Leica Biosystems, Newcastle, UK) [37].

### 4.3. Migration Assay

HASMCs were seeded onto 8-well culture slide (3 × 10^3^ cells/200 μL/well). Cells were incubated at 37°C in 5% CO_2_ for 3–5 h in SmGM-2, and then incubated for 24 h in serum-free SmGM-2 for starvation. Subsequently, while cells were incubated for 16 h in serum-free SmBM with 500 nmol/L angiotensin II and the indicated concentrations of vaspin, photographs of cells were taken for the last 5 h at 10-min intervals. The average migration distance of 10 cells randomly selected in each well was measured using a BIOREVO BZ-9000 microscope (Keyence, Osaka, Japan) [37,38,39,46,47,48,49,50].

### 4.4. Proliferation Assay

HASMCs were seeded onto 96-well plates (1 × 10^4^ cells/100 μL/well) and incubated at 37 °C in 5% CO_2_ for 24 h in SmGM-2 (Lonza). Cells were further incubated for 48 h with the indicated concentrations of vaspin with renewal of each medium. Then, 10 μL of WST-8 solution (Cell Count Reagent SF; Nacalai Tesque, Kyoto, Japan) was added to each well [37,38,39,46,47,48,49,50]. After 1 h of incubation, the amount of formazan product was determined by measuring the absorbance at 450 nm using a Sunrise Remote R™-micro plate reader (Tecan, Kawasaki, Japan) [37,38,39,46,47,48,49,50].

### 4.5. Apoptosis Assay

HASMCs were seeded into 12-well plates (3 × 10^5^ cells/1 mL/well) and incubated at 37 °C in 5% CO_2_ for 24 h in SmGM-2, followed by a 48-h incubation with the indicated concentrations of vaspin. Cells were fixed with 4% paraformaldehyde. Terminal deoxynucleotidyl transferase-mediated deoxyuridine triphosphate-biotin nick end labeling (TUNEL) staining was then performed using an In Situ Apoptosis Detection Kit (Takara Bio, Kusatsu, Japan) as described previously [39,47,48,49].

### 4.6. Foam Cell Formation Assay

THP1 monocytes were seeded onto 3.5-cm dishes (1 × 10^6^ cells/1 mL/dish). Cells were incubated at 37°C in 5% CO_2_ for 3 days in RPMI-1640 medium (Sigma) supplemented with 10% fetal bovine serum, 0.05 mg/mL streptomycin, and 50 U/mL penicillin (monocyte/macrophage conditioning medium (MCM)) and the indicated concentrations of vaspin in the presence of 150 ng/mL phorbol 12-myristate 13-acetate to induce differentiation into macrophages [47,48]. After that, THP1 monocyte-derived macrophages were incubated for 3 days in MCM with vaspin, and further incubated for 2 days in fresh MCM supplemented with vaspin, 50 μg/mL human oxidized LDL, and 100 μmol/L [^3^H]oleate (PerkinElmer, Yokohama, Japan) conjugated with bovine serum albumin [47,48]. Cellular lipids were extracted and the radioactivity of cholesterol-[^3^H]oleate was determined by thin-layer chromatography.

### 4.7. Western Blotting

Aliquots of protein extracts (20 μg) derived from THP1 monocytes, derived macrophages, HASMCs, HUVECs, and HAECs were separated by 10% sodium dodecyl sulfate-polyacrylamide gel electrophoresis, and then immunoblotted with specific antibodies raised against vaspin, and others as described previously [37,38,39,46,47,48,49,50].

### 4.8. Administration of Vaspin into Mice

Animal experiments were performed in accordance with the NIH Guidelines for the Care and Use of Laboratory Animals, with protocols approved by the Institutional Animal Care and Use Committee of Tokyo University of Pharmacy and Life Sciences (No. L15-08, 18 May 2015). A total of 25 male *Apoe*^−/−^ mice (BALB/c. KOR/StmSlc-*Apoe^shl^* mice) at the age of 9 weeks were purchased from Japan SLC and maintained on a normal diet until 13 weeks of age. Subsequently these mice were fed a high-cholesterol diet containing 1.25% cholesterol, 3.0% lard, and 1.625% glucose (Oriental Yeast, Tokyo, Japan). At 17 weeks of age, 7 mice were sacrificed as pre-infusion controls. The remaining 18 were divided into 3 groups of 7, 5, and 6 mice, which were continuously infused with 3 doses of vaspin (0, 50, 100 μg/mouse, respectively) for a period of 4 weeks using osmotic mini-pumps (Alzet Model 1002; Durect, Cupertino, CA, USA). The average dosing rate of continuous infusion was calculated as 0, 2.5, 5 μg/kg/h, respectively. Once every 2 weeks, the mini-pumps were implanted subcutaneously into the dorsum under medetomidine-midazolam-butorphanol anesthesia [48].

At the experimental endpoint (before and 4 weeks after infusion), the *Apoe*^−/−^ mice were sacrificed by exsanguination (total blood collection) under medetomidine-midazolam-butorphanol anesthesia [48]. The whole aorta was immediately washed by perfusion with phosphate buffered saline and fixed with 4% formaldehyde. The aorta was excised from the aortic sinus to the abdominal area, and connective and adipose tissues were carefully removed [37,38,39,47,48,49,50].

### 4.9. Animal Measurements

Body weight and food intake were measured through the study. Systolic and diastolic blood pressures were measured using the indirect tail-cuff method (Kent Scientific, Torrington, CT, USA). At the experimental endpoint, blood samples were collected after a 4-h fast. Plasma levels of glucose, total cholesterol, triglyceride, and free fatty acid were measured by enzymatic methods (Denka Seiken, Tokyo, Japan) [37,38,39,47,48,49,50]. Non-HDL cholesterol was calculated as total cholesterol minus HDL cholesterol. Plasma insulin level was measured by enzyme-linked immunosorbent assay (Ultra-sensitive mouse insulin ELISA kit, Morinaga, Yokohama, Japan) [48]. The HOMA-IR was calculated as fasting plasma insulin (pmol/L) × 0.139 (conversion to μU/mL) × fasting plasma glucose (mg/dL) / 405 [48].

### 4.10. Assessment of Atherosclerotic Lesions

The entire aorta lumen surface and cross-sections of the aortic sinus were stained with Oil Red O for assessment of atherosclerotic lesion area and plaque size, respectively [37,38,39,47,48,49,50]. In aortic sinus wall, monocyte/macrophage infiltration, VSMC content, and vascular inflammation were visualized by staining with antibodies for MOMA2 (Millipore, Burlington, MA, USA), α-SMA (Sigma, St. Louis, MO, USA), or pentraxin 3 (Bioss, Woburn, MA, USA), respectively [37,38,39,47,48,49,50]. The positive stained areas were traced by an investigator blind to the treatment and quantified by image analysis (Adobe Photoshop and NIH ImageJ).

### 4.11. Statistical Analysis

All values are expressed as means ± SEM. The data were compared by the unpaired Student’s *t* test between 2 groups and 1-way analysis of variance followed by Bonferroni’s post hoc test among ≥3 groups using Statview-J 5.0 (SAS Institute, Cary, NC, USA). Statistical significance was defined as *p* < 0.05.

## Figures and Tables

**Table 1:** Characteristics and laboratory data of *Apoe*^−/−^ mice.

Parameter	17 Weeks Old	21 Weeks Old		
	Before	Vaspin 0 μg/kg/h	Vaspin 2.5 μg/kg/h	Vaspin 5.0 μg/kg/h
*N*	7	7	5	6
Body weight (g)	26.2 ± 0.6	28.4 ± 0.5 *	28.7 ± 0.3 *	28.7 ± 0.9 *
Food intake (g/day)	4.0 ± 0.4	4.1 ± 0.1	4.3 ± 0.1	4.1 ± 0.1
Systolic blood pressure (mm Hg)	93.3 ± 1.6	92.1 ± 1.2	89.5 ± 0.2	91.6 ± 2.5
Diastolic blood pressure (mm Hg)	70.3 ± 1.2	71.2 ± 1.6	68.4 ± 0.9	70.4 ± 2.5
Total cholesterol (mg/dL)	2282.3 ± 167.8	2209.7 ± 126.5	2036.8 ± 119.0	2176.5 ± 44.6
Non-HDL cholesterol (mg/dL)	2269.9 ± 168.5	2198.0 ± 125.2	2030.6 ± 118.2	2168.3 ± 44.8
HDL cholesterol (mg/dL)	12.5 ± 2.5	11.7 ± 4.5	6.3 ± 2.0	8.2 ± 1.0
Triglyceride (mg/dL)	287.0 ± 48.8	283.4 ± 38.4	270.8 ± 48.5	174.3 ± 40.7
Free fatty acid (mEq/L)	4.1 ± 0.7	2.7 ± 0.8	2.2 ± 0.5	1.0 ± 0.2 ^†^
Glucose (mg/dL)	295.4 ± 49.0	259.3 ± 31.3	281.3 ± 18.9	172.9 ± 32.3
Insulin (pmol/L)	24.5 ± 7.4	26.2 ± 6.6	65.4 ± 16.4 ^‡^	26.6 ± 10.4
HOMA-IR	2.3 ± 0.6	3.5 ± 1.1	6.6 ± 1.8 ^†^	1.8 ± 0.8

Measurements of body weight, food intake, systolic and diastolic blood pressures, fasting plasma parameters were performed before (17 weeks old) and after 4-week infusion of 3 dosing rates of vaspin (21 weeks old) in *Apoe*^−/−^ mice. * *p* < 0.05, ^†^ *p* < 0.005 vs. 17 weeks old; ^‡^ *p* < 0.05 vs. others.

## Abbreviations

ABCA1	ATP-binding cassette transporter A1
ABCG1	ATP-binding cassette transporter G1
ACAT1	Acyl-coenzyme A: cholesterol acyltransferase 1
CAD	Coronary artery disease
ECM	Extracellular matrix
ER	Endoplasmic reticulum
HAEC	Human aortic endothelial cell
HASMC	Human aortic smooth muscle cell
HOMA-IR	Homeostasis model assessment of insulin resistance
HUVEC	Human umbilical vein endothelial cell
MCM	Monocyte/macrophage conditioning medium
MMP	Matrix metalloproteinase
NCEH	Neutral cholesterol ester hydrolase
NFκB	Nuclear factor κB
LDL	Low-density lipoprotein
PI3K	Phosphoinositide 3-kinase
PPARγ	Peroxisome proliferator-activated receptor γ
ROS	Reactive oxygen species
SRB1	Scavenger receptor class B type 1
VSMC	vascular smooth muscle cell
